# Concurrent white matter bundles and grey matter networks using independent component analysis

**DOI:** 10.1016/j.neuroimage.2017.05.012

**Published:** 2018-04-15

**Authors:** Jonathan O'Muircheartaigh, Saad Jbabdi

**Affiliations:** a Department of Neuroimaging, Institute of Psychiatry, Psychology and Neuroscience, King's College London, London SE5 8AF, United Kingdom; b Centre for the Developing Brain, Division of Imaging Sciences and Biomedical Engineering, St. Thomas’ Hospital, King's College London, London SE1 7EH, United Kingdom; c Oxford Centre for Functional MRI of the Brain, University of Oxford, Oxford OX3 9DU, United Kingdom

## Abstract

Developments in non-invasive diffusion MRI tractography techniques have permitted the investigation of both the anatomy of white matter pathways connecting grey matter regions and their structural integrity. In parallel, there has been an expansion in automated techniques aimed at parcellating grey matter into distinct regions based on functional imaging. Here we apply independent component analysis to whole-brain tractography data to automatically extract brain networks based on their associated white matter pathways. This method decomposes the tractography data into components that consist of paired grey matter ‘nodes’ and white matter ‘edges’, and automatically separates major white matter bundles, including known cortico-cortical and cortico-subcortical tracts. We show how this framework can be used to investigate individual variations in brain networks (in terms of both nodes and edges) as well as their associations with individual differences in behaviour and anatomy. Finally, we investigate correspondences between tractography-based brain components and several canonical resting-state networks derived from functional MRI.

## Highlights

• Parcellation of whole brain grey matter based on diffusion tractography using ICA.
• Data driven patterns of connectivity correspond to region-of-interest based tractography.
• Both hard and soft parcellations show good split-half reliability.
• Cortical and subcortical parcels correspond to known resting state networks.
• ICA provides a principled data-reduction step for tractography data.

## Introduction

Brain mapping has historically tended to focus on local morphological features such as cyto- or myelo-architectonic information, gleaned from post-mortem cortical histology (Amunts and Zilles, 2015). However, the pattern of how brain regions connect macroscopically is increasingly important, and non-invasively tractable, using techniques such as diffusion or functional MRI (Passingham et al., 2002). Anatomical investigations of the human brain using diffusion MRI especially has helped describe its distributed network properties as well as characterise the white matter connections linking these networks (e.g. Ffytche and Catani, 2005; Hagmann et al., 2007).

Defining cortical regions and their connectivity based on MRI is challenging but has been extremely informative (Eickhoff et al., 2015). Non-invasive diffusion tractography has demonstrated marked shifts in connectivity patterns between adjacent regions of tissue, which have been used to inform parcellation of cortical and subcortical grey matter (Anwander et al., 2007, Johansen-Berg et al., 2004, Thiebaut de Schotten et al., 2014). These shifts in the patterns of structural connectivity are reflected in changes in function (e.g. Beckmann et al., 2009; O’Muircheartaigh et al., 2015) and, in a neat proof-of-principle, can predict inter-individual differences in the spatial representation of functional responses to different visual categories (Osher et al., 2016). Connectivity fingerprint-based approaches can also facilitate comparisons between humans and non-human primates, allowing a coarse link between the extensive and detailed invasive animal work and in vivo human investigations (Mars et al., 2016).

There are many approaches for clustering functional regions based on connectivity profiles, both data-driven and based on anatomical prior information (Behrens and Johansen-Berg, 2005). However, for probabilistic white matter tractography data in particular, spatial independent component analysis (ICA) is an intuitive way to provide anatomically meaningful parcellations (Wu et al., 2015). ICA has been traditionally applied to resting-state functional MRI data to extract large-scale networks displaying temporal coherence, so called resting-state networks. ICA has only rarely been used on tractography data. In contrast to most methods, it provides a soft parcellation of the brain, i.e. a weighted assignment of how representative the connectivity patterns of a voxel is of a spatial component (O’Muircheartaigh et al., 2015), and thus different connectivity components can substantially overlap in space. Spatial components calculated using ICA have been shown to reflect major white matter bundles and the parcels themselves are functionally specific and may also be sensitive to subtle changes in disease (O’Muircheartaigh et al., 2012, Wu et al., 2015). Depending on where independence is enforced, the patterns of connectivity driving an ICA solution can be spatially independent in seed space (e.g. the regions from which tractography is seeded, usually grey matter) or in tract space, depending on which is most appropriate.

Additionally, ICA provides a single framework to identify both the parcels (the seeds for tractography/nodes) and the white matter tractography connections (edges). In this study, we use spatial ICA to explore whole brain tractography connectivity data in multiple subjects. We propose an approach to group ICA of tractography data that allows efficient, accurate, and scalable analysis of very large connectivity data sets in an unsupervised way (though dimensionality must be specified *a priori*). We provide multi-scale parcellations of cortex based on this approach in both group average and single subject analyses and we demonstrate the reproducibility of these ICA decompositions using split-half analyses. Further we investigate the anatomical relevance of the IC maps both cortically and in the white matter. To this aim we compare the white matter components to traditional virtual tract-dissection approaches and the associated gray matter components to functional networks obtained through ICA of resting-state FMRI data. Finally, we give a proof-of-concept use of our group ICA approach to tractography to characterising inter-individual variation in connectional neuroanatomy.

## Methods

### Participants and datasets

**Fig. 1:**
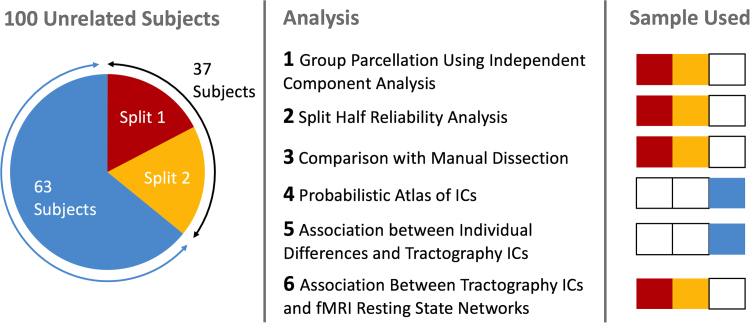
Summary plot of the datasets used in this study and a breakdown of which datasets were used in each of the analyses conducted.

Data were made available by the Human Connectome Project (humanconnectome.org). Of the available data, structural T_1_-weighted, diffusion-weighted and functional BOLD-weighted MRI data were downloaded from the Human Connectome Project database (http://www.humanconnectome.org). In total 100 datasets were included for this study. For the initial parcellation and the split-half analysis, the “40 unrelated subjects” dataset was used, with a total of 37 useable subjects (20 female) all aged between 22 and 35 years. For the exploratory study investigating individual differences, an additional 63 subjects from the “100 unrelated subjects” dataset were used (34 female, same age range). See Fig. 1 for a summary breakdown of which datasets were used in each analysis.

### MRI data acquisition

MRI data were acquired at Washington University St Louis using a Siemens Magnetom Connectome 3 T scanner. Acquisition and basic spatial preprocessing protocols are described in detail elsewhere (Andersson and Sotiropoulos, 2016, Sotiropoulos et al., 2013, Uğurbil et al., 2013) and spatial preprocessing of the anatomical MPRAGE T_1_-weighted image (FOV=224×224 mm, matrix=320, 256 sagittal slices, TR=2400 ms, TE=2.14 ms, TI=1000 ms, FA=8°, 0.7 mm isotropic resolution) are described in detail in Glasser et al. (2013). Diffusion MRI data consisted of 3 shells (b-values=1000, 2000, and 3000 s/mm^2^) with 270 diffusion directions equally spread amongst the shells, and six b=0 acquisitions within each shell (FOV=210×180 mm, matrix=168×144, 111 axial slices, TR=5520 ms, TE=89.5, isotropic spatial resolution of 1.25 mm). In addition, for each subject, 4 runs of 15 min of resting state fMRI were collected (FOV=204×108 mm, matrix=104×90, 72 axial slices, TR=0.72 s, TE=0.33 ms, FA=52°, 2 mm isotropic resolution, 1200 time points per run).

### Diffusion data preprocessing and tractography

Subcortical masks and cortical surfaces were calculated using the Freesurfer package as described in Glasser et al. (2013) and all analyses were performed in MNI space. Each dataset was prepared for probabilistic tractography using the bedpostX algorithm, modified to account for multi-shell acquisitions, assuming a Rician noise model (Jbabdi et al., 2012), and modelling for up to three fibre populations per voxel. The probtrackx2 programme was used for the tractography itself (Behrens et al., 2007, Hernández et al., 2013). Probabilistic tractography was performed by seeding in standard MNI space (2 mm resolution) from each of 9127 subcortical voxels (bilateral thalamus, caudate, putamen, pallidum, amygdala, hippocampus and nucleus accumbens) and from each of 59,412 cortical vertices (seeded from the mid grey matter surface, excluding the non-cortical medial wall), with 5000 streamline samples initiated from each voxel/vertex. Cerebellar and brainstem regions were not used as seeds in this analysis. In addition, stopping masks were specified on the cortical pial surface, to avoid the possibility of erroneous cross-sulcal fibres, and at the ventricles. The resulting dataset for each subject consisted of a connectivity matrix of streamline visitation counts for each of 68,539 seed regions to 80,090 possible targets voxels (all voxels of the MNI brain downsampled to 3 mm isotropic). Visitation counts were multiplied by the expected length of the tracts at each voxel to compensate for the distance bias (longer tracts tend to have higher compounded uncertainty and therefore lower visitation counts).

### Independent component analysis of tractography data (Fig. 2)

**Fig. 2:**
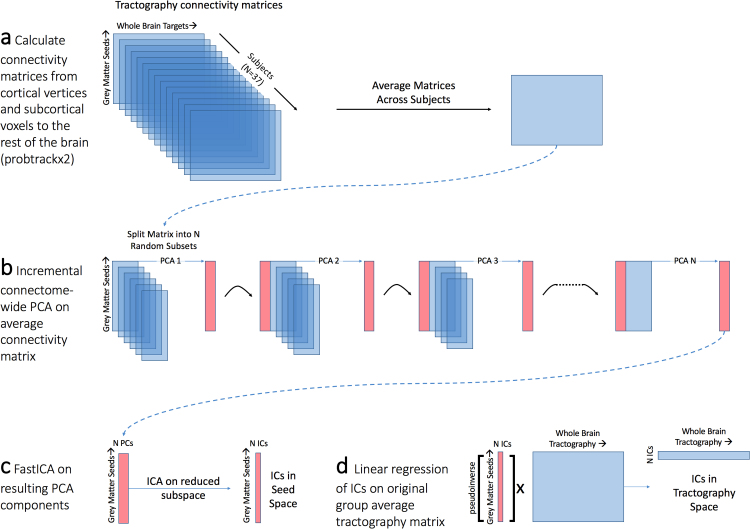
Independent Component Analysis on tractography matrices. (a) Probabilistic tractography is performed in 37 subjects, seeded from each cortical vertex and subcortical voxel in the brain to the rest of the brain. This matrix is averaged across subjects to provide a group-average connectivity matrix. (b) The dimensionality of this matrix is incrementally reduced in *tractography space* using principal component analysis on subsets of the matrix. (c) Independent component analysis is performed on this reduced matrix providing independent components in seed space but without a straightforward mapping to tractography. (d) GM maps are projected back onto the group average tractography matrix using linear regression providing a tractography representation of the independent components.

Here, by tractography data, we mean a matrix with dimensions (number of GM voxels/vertices)×(number of WM voxels), where each entry corresponds to the number of streamlines running from a grey matter position to a white matter location (weighted by the average distance from the seed). Because each row of the matrix corresponds to a single seed voxel, the matrix can be re-written as a sum of outer-product components, where each component is a vector of white matter pathways multiplied by the vector encoding the corresponding seed voxel (Fig. 2a). If two seed voxels have the same WM connectivity, the rank of the matrix drops by one. Therefore, both principal component analyses and independent component analyses will tend to group together seed voxels with similar WM fingerprints. We first apply PCA to reduce the dimensionality of the data matrix, followed by ICA to relax the orthogonality constraints of PCA, allowing e.g. spatial overlap between WM components.

For the group analysis, the tractography data matrices were first averaged across 37 subjects. This initial average representative matrix was then reduced in size using principal component analysis (PCA). As this dataset was very large (68,539 seed voxels against a volume of 80,090 possible target voxels), PCA was performed using an adapted incremental method referred to as Melodic's Incremental Groupwise PCA (MIGP) in Smith et al. (2014) and performed in MATLAB, though on the group-average connectivity matrix instead of a concatenation of individual subjects (Fig. 2b).

In brief, for each iteration of PCA, a matrix of all cortical and subcortical seeds against a random subset (10,000) of whole brain target voxels were reduced to 4000 PCs, then a different random subset of 10,000 whole brain voxel values were concatenated to these 4000 eigenvectors (weighted by their corresponding eigenvalues) and PCA was run on this combined matrix, and so on until the whole brain has been covered. At each iteration, only a relatively small matrix (68,539×10,000) is analysed with PCA, and thus this approach can be applied to approximate a PCA of very large matrices (Smith et al., 2014). The fastICA algorithm (Hyvarinen, 1999), implemented in Matlab (available at http://research.ics.aalto.fi/ica/fastica/) was then applied to the resulting reduced dataset (Fig. 2c). We performed ICA across a series of dimensionalities (K=50, 100, 150, 200, 300) with independence enforced in the *seed domain*, thus grey matter components were statistically independent from each other, whereas the spatial distributions of white matter components could overlap.

This resulted in a set of K spatially independent maps in grey matter cortical/subcortical regions with K associated spatial tractography fingerprints. As these patterns were represented in the PCA subspace only (i.e. keeping 4000 dimensions instead of the full 80,090), the normalised weighted ICs were projected back onto the full average tractography connectivity matrix using linear regression to reconstruct the whole brain tractography connectivity pattern (Fig. 2d). The resulting components in seed space and their white matter counterparts were fitted to a Gaussian/gamma mixture model as in (Beckmann, 2012), with the positive gamma distribution thresholded at p>0.5. In addition, for each dimensionality, the cortical surface was parcellated according to which component had highest weighting in each vertex (i.e. winner-take-all), providing a hard parcellation of the cortical surface. Example matlab code to perform this is included in the Appendix. Finally, we also performed the same analysis using individual subject tractography connectivity matrices.

### Comparison with the virtual dissection approach

To investigate the anatomical relevance of the white matter tractography ICA components, we compared them to the results of a more traditional virtual dissection approach. We used a fully automated probabilistic tractography approach (de Groot et al., 2013) which extracts 13 major tracts bilaterally (Supplementary Table 1) based on an a priori set of inclusion/exclusion/seed masks, defined in MNI152 standard space. The same 37 subjects were included in this analysis and the patterns of connectivity were averaged across subjects as in the group ICA analysis. Scripts and masks are freely available for download (http://fsl.fmrib.ox.ac.uk/fsl/fslwiki/AutoPtx). Similarity of the results of ICA and virtual dissection were assessed using spatial correlation coefficient (Pearson's r).

### Split-half reliability analysis

To assess the overall reliability of the resulting ICs, we split the group of 37 into two equally sized groups of 18 (discarding one subject for this analysis) and ran ICA with dimensionalities in steps of 25 from K=25 to K=250. For each split-pair, Pearson correlation coefficients were calculated between each of the component weights and their corresponding component (the component from the other split with the highest absolute correlation coefficient). Similarly, the Dice coefficient was used to assess reproducibility of hard parcellations between each split of subjects for each dimensionality (Parisot et al., 2015, Parisot et al., 2015). This analysis was performed on the cortical surface only and compared to the Dice coefficients of 1000 random parcellations at the same dimensionalities. Using ICA, spatial contiguity is not explicitly enforced, so the dice coefficient can be calculated between sets of non-contiguous regions.

### Assessing individual variation in tractography independent components and their association with individual differences

We investigated individual differences in IC maps and their relationship with demographics. To this end, we needed to first obtain individual subjects’ versions of the group ICA results. For this we used regression of the group ICA results onto single subject matrices as performed commonly in fMRI (Filippini et al., 2009). In this regression, the design matrix is a group map (the group ICA maps) and single subject tractography matrix is the data. The resulting regression coefficients approximate single subject versions of the group maps. We apply this method with both the cortical (node) and tractography (edge) maps from our group ICA as the design matrices (with k=50) providing their complement for each subject. This provided single subject maps of the node and edges for each group component for the 63 unseen subjects (see Fig. 1).

In addition, we estimated a single value representation of each IC in tractography and grey-matter dimensions by calculating the dot-product of both the group tractography pattern with single subject tractography patterns and the group grey matter IC with the single-subject IC, providing two weights for each IC and subject. Spearman's rank correlations were calculated between both the subject weights of the grey matter IC (nodes) and their tractography representation (edges) and individual differences in (1) age and gender (2) behavioural and cognitive scales (3) tissue volume measures (4) cortical area measures and (5) cortical thickness measures. Measures 3,4 and 5 were calculated from the T1 weighted volume as part of the Human Connectome Project standard preprocessing scheme (Glasser et al., 2013) and all variables used are attached as Supplementary Table 2. Corrections for the resulting 26,300 multiple comparisons were performed using false discovery rate across all tests, fixed at q<0.05, and implemented in Matlab using the Storey (2002) approach (function mafdr.m).

### Relationships between group level tractography ICs and functional resting state networks

Functional resting state networks were also calculated using group independent component analysis using data preprocessed with the FMRIB ICA-based Xnoiseifier (FIX; Salimi-Khorshidi et al., 2014) and released as part of the HCP (Smith et al., 2013). Four runs of resting state fMRI for each subject (37 total, the same sample as used in the initial ICA used on tractography data) were input into a group ICA analysis, also using MIGP for the initial PCA step (Smith et al., 2014), after high pass filtering the timeseries with a 100 s filter. The number of independent components was set to 21 and these resulting functional networks were categorised according to their similarity with previously published resting state networks (Smith et al., 2012).

Structure-function correspondence between resting state networks, derived from ICA on fMRI data, and the 50 dimensionality tractography ICA was measured using Pearson's correlation coefficient. As ICA results in a set of components that have minimal co-linearity, we can use the squared correlations between all structural ICs and any given functional network as an indication of percent variance in the spatial organisation of functional networks accounted for by the structural ICs. Given the high dimensionality of the data considered here, we did not perform null-hypothesis significance testing of the structure-function associations as even very weak associations would be statistically significant. Instead we considered relationships significant if the proportion of variance accounted for by any structural IC for each functional IC was higher than 5% (e.g. a spatial correlation between modalities>0.22).

## Results

**Fig. 3:**
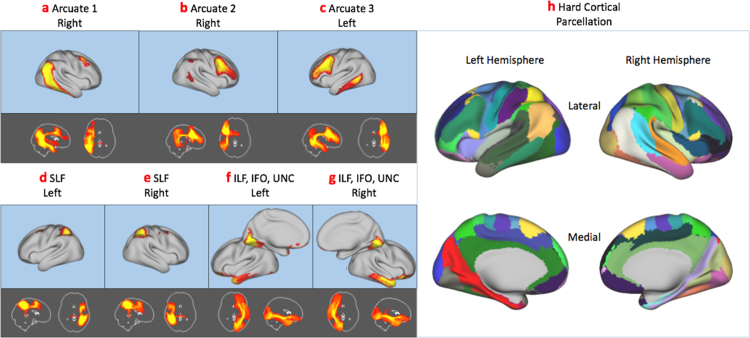
Example cortico-cortical components (top rows) and their patterns of connectivity (bottom rows) taken from the K=50 independent component analysis. Note that some ICs are represented by multiple white matter bundles (e.g. the ventral stream in components f and g). A hard parcellation of the brain based on the ICA weights is shown in h. In this and all figures, the ICs themselves and the patterns of connectivity are thresholded using mixture modelling at p<0.5 (see *Methods*). SLF – Superior Longitudinal Fasciculus, IFO – Inferior Fronto-occipital Fasciculus ILF – Inferior Longitudinal Fasciculus, UNC – Uncinate Fasciculus.

**Fig. 4:**
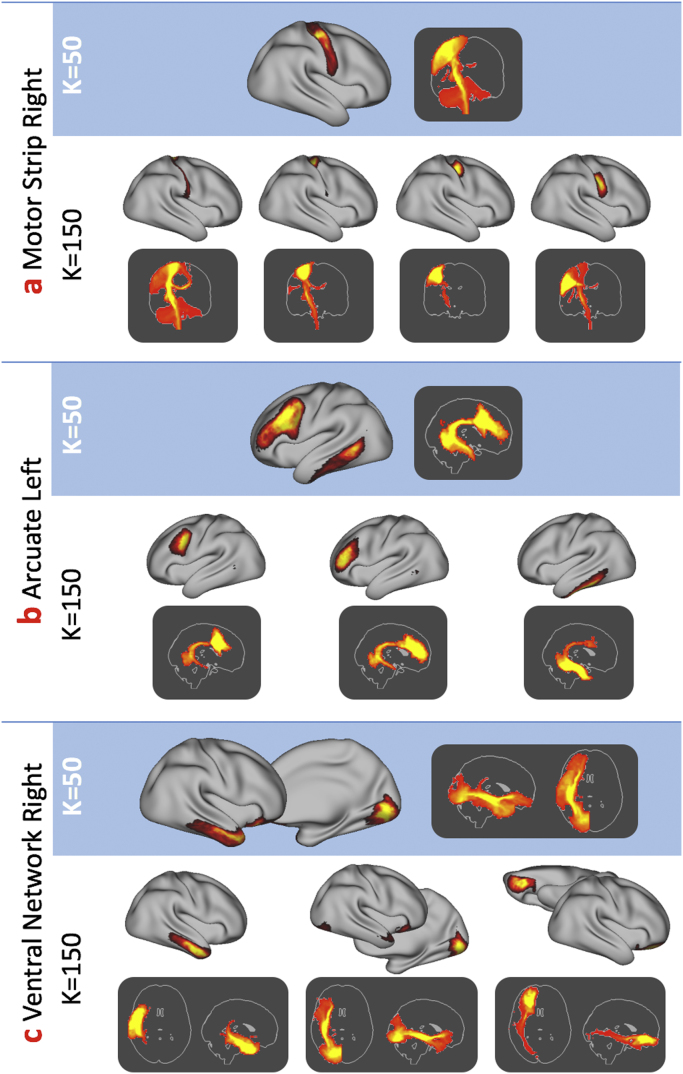
Illustration of the effect of ICA dimensionality selection on resulting components, here comparing components identified in a K=50 ICA with their most similar (highest spatial correlation) equivalent components in the K=150 ICA. In three examples, (a) the motor strip gets split into four neighbouring subregions, (b) grey matter associated with the arcuate into three subregions with slightly different spatial trajectories of structural connectivity and (c) a ventral and lateral network (not associated with any single specific fibre bundle) gets split into three more anatomically distinct and identifiable regions.

Spatial independent component analysis resulted in a series of weighted cortical/subcortical parcels representing consistent spatial *patterns* of white matter connectivity. For a low dimensionality analysis (K=50), anatomically meaningful white matter bundles were evident such as the arcuate and superior longitudinal fasciculi bilaterally (Fig. 3a-e). In addition, *networks* of interconnected regions were evident, such as the inferior fronto-occipital, inferior longitudinal and the uncinate fasciculi (Fig. 3f, g) which course via occipital and ventral frontal cortex as well as the temporal pole. Classifying each cortical vertex according to the IC with the highest weight provided a hard parcellation of cortex (Fig. 3h). Higher dimensionality ICA tended to split individual components into subparts (identified using spatial correlation), such as different regions of motor cortex (Fig. 4a) or splitting the uncinate and inferior longitudinal fasciculus (Fig. 4c). Group cortical hard parcellations at k=50,100,150,200 and 300 are included as Supplementary images 1–5. Raw and thresholded ICs and their tractography patterns for k=50 are included as Supplementary images 6–9.

**Fig. 5:**
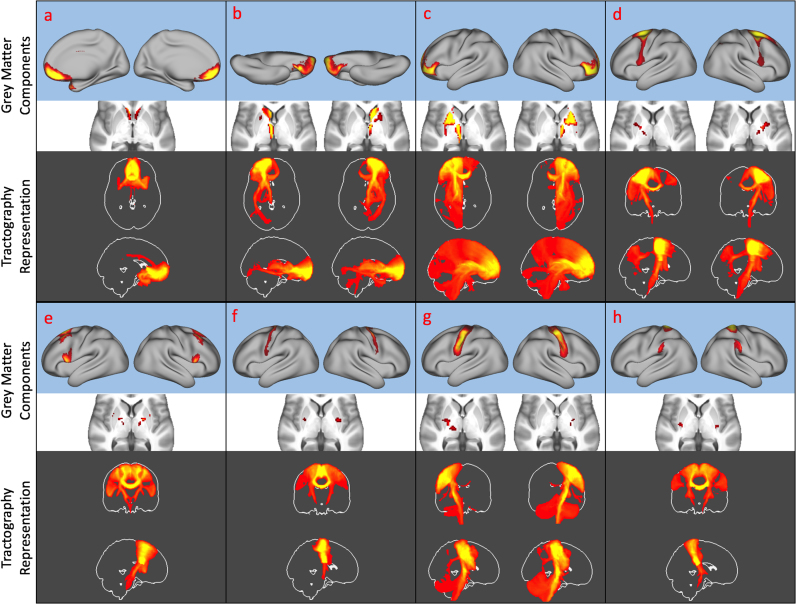
Basal ganglia-cortical loops decomposed using tractography and ICA. The top rows show the cortical (blue background) and subcortical (white background) representation of independent components, following an anterior to posterior gradient from a to h. The corresponding white matter tractography maps are shown as MIP (maximum intensity projection, orange with grey background). Some loops have lateralised components, in this case they are shown with their corresponding contralateral component (b,c,d,g) In all images, left is left.

These patterns of white matter connectivity also clearly delineated grey matter cortical/subcortical networks with appropriate grey matter regions mapping to known thalamo-cortico-striatal loops (Alexander and Crutcher, 1990, Draganski et al., 2008, see Fig. 5). This significantly extends prior work on thalamocortical diffusion connectivity by directly including both cortex and basal ganglia in such loops (e.g. Behrens et al., 2003; O'Muircheartaigh et al., 2015). It also reproduces the matched dorsal to ventral (cortical) and medial to lateral (subcortical) gradients described in track tracing experiments (e.g. Jbabdi et al., 2013).

**Fig. S1:**
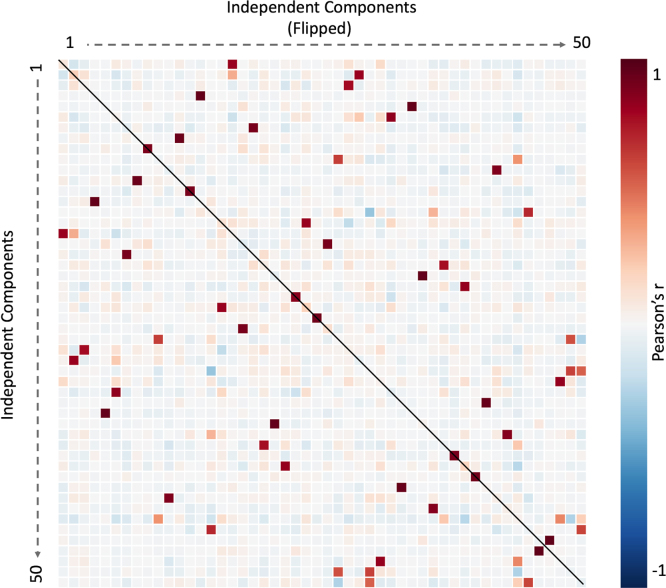
Supplementary Fig 1: Cortical symmetry of components from the k=50 ICA analysis. All components have at least one contralateral homologue, though the strength of similarity varies. Six components are bilateral, as indicated by lying on the diagonal.

These components are also clearly symmetric. Pearson's spatial correlations of the cortical components with their x-axis flipped counterparts are shown in Supplementary Fig. 1. Of the 50 ICs, 6 are bilateral, symmetric (examples are shown in Fig. 5a, e, f, and h). Of the remaining 44, exactly half (22) are left lateralised and half are right lateralised. All have a contralateral homologue with a spatial correlation of r>0.6 and the majority (16/22) of these have contralateral homologues with a spatial correlation of r>0.8 as seen in Fig. 5b, c, d and g.

### Comparison to virtual dissection

**Fig. S2:**
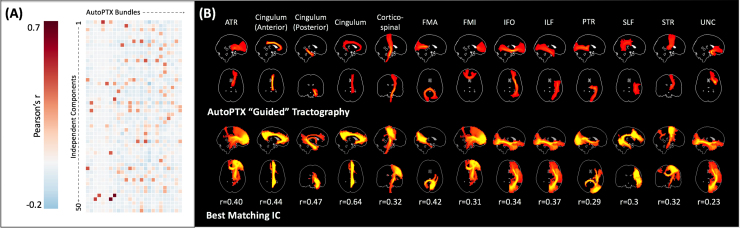
Supplementary Fig 2: Correspondence between ICA-tractography maps (K=50 only) and an alternative automated approach using regions of interest (AutoPTX), calculated on the same 37 subjects. Panel A shows the spatial correlation between each of 26 white matter bundles calculated using AutoPTx (x-axis) and the tractography pattern associated with each IC in the 50 dimensionality ICA (y-axis), performed using the same datasets. Panel B shows the white matter bundles calculated using AutoPTx (top row) and the most correlated IC with that bundle for the left hemisphere only (bottom row). Pearson's correlation coefficients are also shown.

The patterns of connectivity identified using ICA were compared to an automated tractography heuristic (de Groot et al., 2013) and showed that a subset of the ICs matched well spatially and anatomically to known white matter bundles (Supplementary Fig. 2). The tracks identified using the virtual dissection approach are guided by inclusion, exclusion and stop masks, and are therefore strongly driven by anatomical knowledge. Though they are systematically spatially smaller than the tractography ICs, the spatial correspondence is clear.

### Reproducibility

**Fig. 6:**
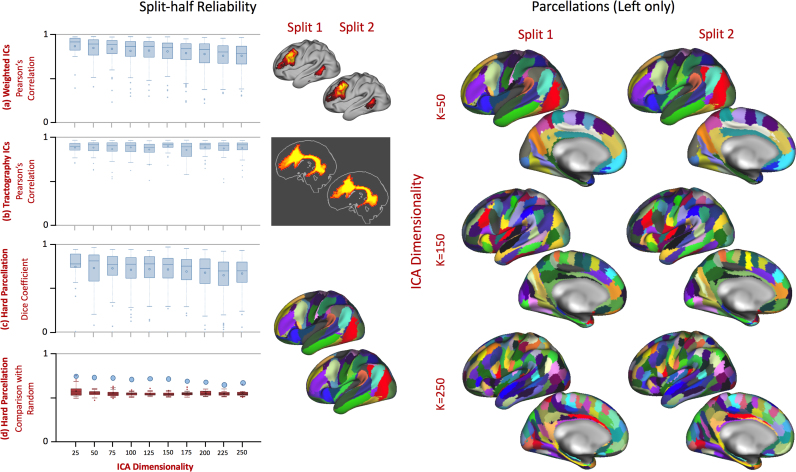
Split-half reliability analysis across ICA dimensionalities. For each dimensionality, the reliability across individual components in one split and their representative alternate in the other split is displayed as a box plot for the weighted grey matter (a) and tractography (b) or categorical parcels (c). The mean dice coefficient (blue dot in plots c and d) of the ICA parcellation is higher than a random Voronoi parcellation (the red boxplots in d represent the range of mean dice coefficient for each of 100 iterations of random parcellations). An example component from the left hemisphere, representing the arcuate fasciculus, is demonstrated for each split. As would be expected from this high reliability, hard parcellations (right panel), shown on the left hemisphere only, are visually similar to each other at different ICA dimensionalities.

After performing ICA on the two splits, the resulting ICs were hard-thresholded to parcellate the brain according to dominant patterns of connectivity. Both the full weighted ICA analysis and the hard parcellation showed good reliability and reproducibility across different dimensionalities in a split-half analysis (Fig. 6). Median correlation across grey matter components, between splits, degraded linearly from 0.9 to 0.78 as dimensionality increased from 25 to 250 components (Fig. 6a) though the white matter tractography representation of each independent component was highly stable between splits (Fig. 6b). Dice coefficients of the hard parcellation decreased from median values of 0.85 to 0.7 (Fig. 6c). Importantly, the mean Dice coefficient for all splits was higher than random parcellations of the same dimensionality, even though these random parcellations explicitly imposed spatial contiguity of parcels (a constraint not imposed in the ICA analysis), and thus were likely to have high dice coefficients (Fig. 6d).

### Projection to individual subjects

**Fig. S3:**
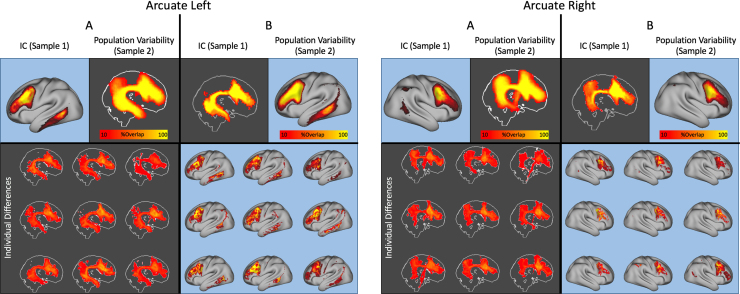
Supplementary Fig 3: Independent components corresponding to the arcuate fasciculi in the left and right hemisphere. For each hemisphere a regression of the cortical independent component (top left of each panels A) gives a consistent reconstruction of the arcuate fasciculus across 63 subjects in an independent sample (top right of panels A for population probability maps) and in 9 example subjects (bottom of panels A). Similarly, a regression of the white matter tractography pattern identified using ICA (top left of panels B) shows a reproducible cortical distribution (top right of panels B for population probability maps) and in 9 example subjects (bottom of panels B). Maps of individual datasets are shown after thresholding to the positive gamma distribution at p>0.5 as described in the *Methods*.

**Fig. S4:**
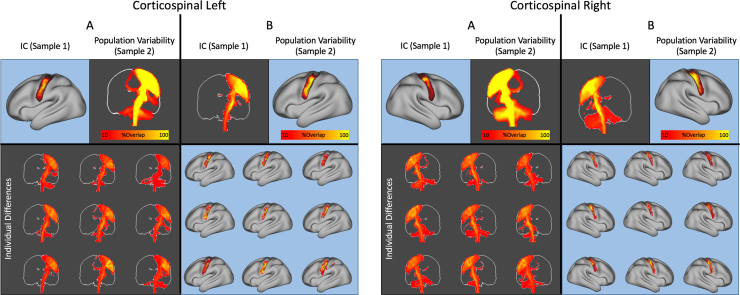
Supplementary Fig 4: Independent components corresponding to the bilateral corticospinal tracts. Panels are arranged as in supplementary figure 3.

**Fig. S5:**
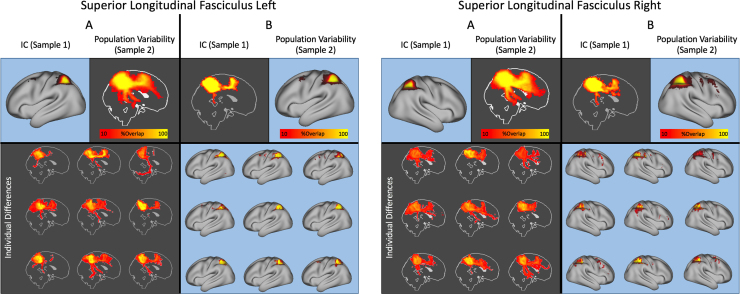
Supplementary Fig 5: Independent components corresponding to the bilateral superior longitudinal fasciculi. Panels are arranged as in supplementary figure 3.

**Fig. S6:**
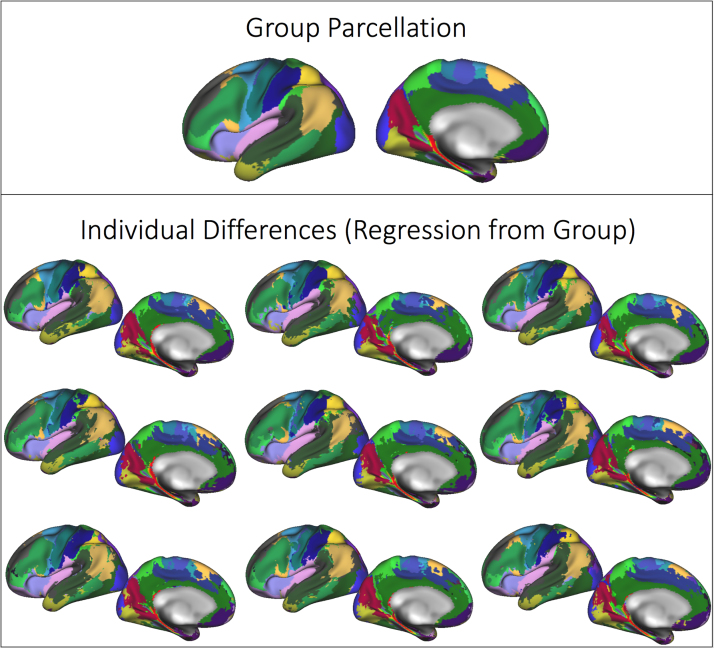
Supplementary Fig 6: Group hard parcellation (top row, dimensionality of 50, left hemisphere only displayed) projected onto nine individual subjects. Patterns of white matter connectivity from the group analysis are regressed onto individual connectivity matrices using linear regression and the grey matter patterns hard thresholded.

We regressed the group independent components and paired tractography patterns onto the connectivity matrices of individual subjects not used in the initial group ICA (n=63, see Fig. 1). This resulted in patterns of cortical/tractography connectivity consistent with the group patterns (see Supplementary Figs. 3–5 for examples of the bilateral arcuate fasciculi, corticospinal tracts and superior longitudinal fasciculi). Across the 63 subjects both the white matter course and grey matter origin of these tracks were stable across subjects but inter-individual variability was evident. Supplementary Fig. 6 demonstrates individual differences in the hard parcellation of nine individual subjects.

**Fig. 7:**
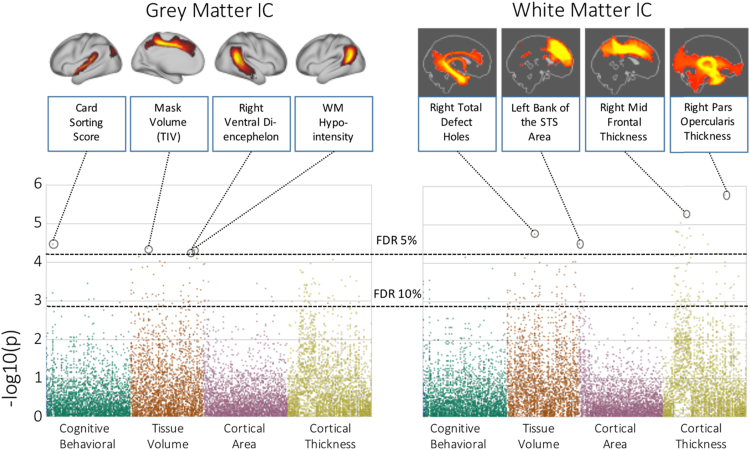
Associations between independent components and 262 different behavioural, cognitive and structural MRI measures (calculated on the T1 weighted volume). Correlations are arranged as a Manhattan plot with the dashed lines indicating the 5% and 10% line for false discovery rate across all correlations.

There were significant associations between individual differences in the representations of these ICs and cognitive and structural brain indices collected as part of the HCP. In total 26,300 tests were performed, though only 23 survived multiple comparison correction using false discovery. The majority of the associations were with measures derived from the structural MRI data, especially regional cortical thickness and tissue volumes (see Fig. 7) and even initial errors in cortical surface calculation (total defects/holes in the right hemisphere).

### Comparison of tractography ICs to fMRI-based resting-state networks

**Fig. 8:**
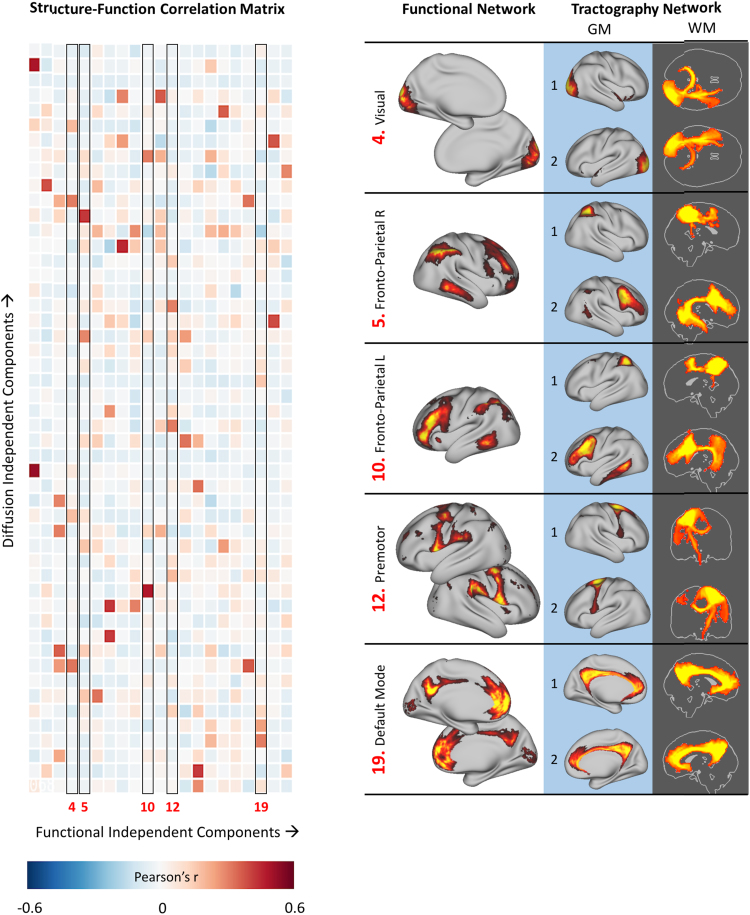
Association between tractography and resting state networks (left panel). In the right panel, five example resting-state fMRI based functional networks (left column) with the two diffusion tractography-based independent components that are most strongly associated with them (middle column), as well as their pattern of connectivity from these components as maximum intensity projections (right column), are shown. Resting state networks shown on the right panel are highlighted in the correlation matrix on the left panel by a black box outline and their column number in the matrix.

Functional networks derived from a temporal concatenation group ICA on the resting-state fMRI data resulted in 21 functional networks, characteristic of those published previously using similar data (e.g. Smith et al., 2013). For all functional networks except one, a clear relationship between tractography ICs and functional networks were apparent (Fig. 8, left panel for the full correlation matrix) and the associated white matter patterns appeared to be anatomically meaningful, with the arcuate and superior longitudinal fasciculi being most associated with fronto-parietal networks on both cortical hemispheres (Fig. 8, right panel). Note that the number of vertices/seeds per correlation is very large (68,539) so even very weak correlation coefficients are significant, so in Fig. 8 (right panel) only the two strongest associations are shown. The correlation matrix is sparse, indicating specificity of the relationships of structural to functional networks. The only functional network that showed no association between structure and function was a resting state network involving the cerebellum, a structure not included in our tractography seeding strategy and representing a true negative.

## Discussion

The specialisation of brain regions and their integration within networks is reflected by their connectivity patterns. Using a data-driven technique, we illustrate cortical and subcortical regions defined by their diffusion tractography-based connectivity fingerprints. In addition to isolated regions, we show *non-contiguous* sets of brain regions that are characterised by common pathways of connectivity both to each other and to the rest of the brain, replicating anatomically driven manual tractography studies. This approach is analogous to earlier track tracing work in animals (Selemon and Goldman-Rakic, 1988) where work has aimed to functionally link spatially disparate cortical regions according to their whole brain connections. Through parcelling cortical and subcortical regions in a data-driven and soft fashion, we provide both weighted and hard atlases of structural brain connectivity that concurrently address both the topological and hodological nature of brain connectivity.

**Fig. S7:**
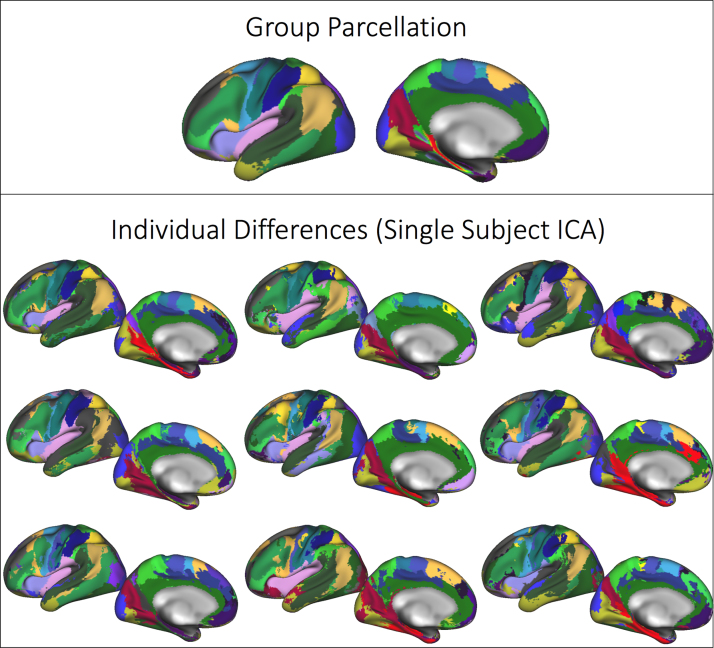
Supplementary Fig 7: ICA performed on individual subject connectivity matrices (bottom row, dimensionality of 50, left hemisphere only displayed). Cortical parcels are colour coded according to the group ICA (top row) tractography component to which they are most spatially similar, though the group analysis is otherwise independent.

The connectivity profiles demonstrated here are anatomically meaningful. In the absence of a constraint of spatial contiguity, this method determines known cortico-cortical pathways and subcortical-cortical loops without prior anatomical information or manual intervention beyond specifying the number of components (Fig. 3, Fig. 5). The patterns are symmetric. Lateralised components have contralateral homologues (Supplementary Fig. 1) with some being bilaterally symmetric. Interestingly the component corresponding to the arcuate fasciculus (Fig. 3a, b and c) is asymmetric in their temporal terminations especially, replicating prior work (e.g. Glasser and Rilling, 2008; Takaya et al., 2015). Though this is not a validation of the parcellation as such, it does provide some face validity. As well as being reproducible on a group level (Fig. 6), the patterns are grossly reproducible when ICA is performed in single subjects with no prior constraints (Supplementary Fig. 7).

As expected from Wu et al. (2015), increasing ICA dimensionality leads to a finer delineation of cortical regions (Fig. 4), and in some cases splitting bilateral components into lateralised components. Fig. 4c, in particular, was selected specifically to replicate the findings in this work. In addition, using simple linear regression of the tractography patterns from the group ICA, our approach provides a way of projecting population averaged parcels and white matter pathways (weighted or binary) back to individual subjects (Supplementary Figs. 3–5). The parcellations are also functionally meaningful. Our results associating structural tractography connections to functional resting state networks replicate and extend manually delineated tractography studies (e.g. Greicius et al., 2008; van den Heuvel et al., 2009).

In connectivity analyses (functional or structural), the choice of seed regions/nodes is critical. True regional and population heterogeneity in functional and structural architecture combine with parcellation dimensionality to have a strong influence on resulting network properties (de Reus and van den Heuvel, 2013, Smith et al., 2011, Zalesky et al., 2010). Though functional parcellations defined by functional homogeneity, regional timeseries correlation or some other temporal feature, can be useful, these parcellations can change according to attentional or cognitive state. For example, in Laumann et al. (2015), functional MRI based parcellations using a community detection approach showed differential boundaries in the occipital lobe *of the same subject* depending on whether their eyes were open or not.

This variability should be expected; functional co-activity (and therefore resulting connectivity-based parcellations) in fMRI reflects context/cognitive state. Indeed, this is why it is such an important tool in cognitive neuroscience. However, this may make functional measures of brain connectivity less appropriate for identifying *consistent* parcellations of cortical areas without explicit priors guided by inter-individual variability (e.g. Wang et al., 2015). A clear advantage of a parcellation guided by structural connectivity is that it may be used to illustrate how different fixed overlapping regional pathways get co-opted by different cognitive functions (Jbabdi et al., 2015, Osher et al., 2016, Park and Friston, 2013).

Functional networks defined by resting state fMRI interact over a longer time scale than that which would be expected by mono- or multi-synaptic white matter connections that may be inferred from diffusion tractography (Petersen and Sporns, 2015). Above the thalamus, white matter structural connections are made up of lateralised association and projection bundles as well as commissural bundles which are bilateral and exhibit gross left-right symmetry (Catani and Thiebaut de Schotten, 2008) whereas there is no such natural constraint on fMRI-based functional connectivity. The relationships we illustrate between white matter ICs and functional networks reflect this mismatch, with combinations of different white matter projections *cumulatively* explaining the spatial distribution of functional networks (Fig. 8). The mismatch between methods is further confounded by possibly non-neural functional connectivity that can be described by population variation in vasculature, metabolic rate, ageing etc. (Murphy et al., 2013). Through looking at a population average of high quality data, collected within a relatively tight age-range, we minimise these effects. So for each fMRI component we were able to identify multiple tractography components that collectively contribute to their full spatial pattern (Fig. 8).

A single decomposition of both grey and white matter provides the opportunity to investigate brain injury or disease in a more natural way. Injury to spatially distributed white matter and grey matter regions can lead to similar cognitive and neurological outcome(Boes et al., 2015; Corbetta et al., 2015). An approach such as this, which can quantify injury to grey or white matter as being associated clearly with the interruption of one or many structural networks (whether node or edge) in place of a region, can potentially provide higher sensitivity compared to voxel-based techniques that rely on spatial overlap (Griffa et al., 2013). We have previously taken this approach in generalised epilepsy (O’Muircheartaigh et al., 2012) and Wu et al. (2015) have applied it in schizophrenia, using multivariate weights of tractography-based independent components and using them as dependent variables. In clinically variable diseases and injuries such as multiple sclerosis and stroke, this type of approach may also provide a good link between grey matter atrophy and white matter injury (Filippi et al., 2012).

Another main advantage is that the number of statistical comparisons can be greatly reduced. In a proof of concept, we investigated the association between the weights of tractography ICs from the K=50 independent component analysis and their associations with over 250 individual differences in behavioural, cognitive and structural measures. A small subset of associations survived false discovery rate correction (23 of 26,300 tests). Individual differences in the representation of the grey matter components and their white matter tractography patterns were mostly associated with regional grey matter volume and thickness (Fig. 7). Larger studies (e.g. Miller et al., 2016) have clearly demonstrated that associations between behaviour, cognition and MRI measures can be weak in a normal population (i.e. with no obvious pathology) and we would need significantly larger samples to detect associations between brain measures and these individual difference, especially in healthy adults (Gignac and Szodorai, 2016).

Clearly, there are number of reasons to be cautious in the interpretation of any measure based on tractography. Firstly, there is the obvious caveat that probabilistic tractography does not directly quantify white matter integrity, but rather our uncertainty on streamlines through the diffusion field (Jbabdi and Johansen-Berg, 2011). Additionally, by using a group average of connectivity matrices, we are effectively creating a sample-specific template of how the brain is connected. Individual differences in connectivity are therefore relative to this template. For the same reason, registration plays an important further role, as individual differences in registration accuracy (in both gray and white matter) will clearly affect the results. An example of this may be seen in Fig. 7. There was a significant association between the tractography IC corresponding to the posterior cingulum, as it leads into the mesial temporal lobe, and topological defects in surface extraction, which can occur in the medial temporal lobe especially, so this may reflect true morphological changes. Spatial smoothing in either seed or target space may help reduce the effect of subtle mis-registration (O’Muircheartaigh et al., 2015), as may explicitly including brain connectivity data as part of the registration cost-function (e.g. Robinson et al., 2014).

A more obvious constraint that we chose, specific to this analysis, is on which dimension to impose independence. In fMRI, this is a choice between spatial and temporal independence for resulting components. In this application in tractography, the choice is between imposing independence in the seed domain (grey matter vertices/voxels) or the tractography domain (presumed white matter connectivity). Previous work, including our own, has imposed independence in tractography space (O’Muircheartaigh et al., 2011). This was specifically to segregate thalamocortical fibres which, at typical diffusion MRI resolution, mostly follow a single, non-overlapping course. However, this is a simplification and is certainly not the case for most of the brain, where multiple fibre populations are the norm (e.g. Jeurissen et al., 2013), even at the relatively high spatial resolution of the data used here. Imposing independence on the tractography domain led to very localised parcels of white matter with distributed grey matter end points, in this case representing areas in (for example) frontal white matter through which multiple white matter pathways course but are not part of any single bundle.

ICA is just one possible approach to parcelling both grey and white matter. Another approach, also working in grey matter space, by Thiebaut de Schotten et al., 2014, Thiebaut de Schotten et al., 2016 utilised principal component analysis to parcel occipital cortex and frontal cortex respectively, providing anatomical regions defined by orthogonal patterns of connectivity, though the patterns themselves rarely corresponded to just one anatomical bundle (as is the case here at low model order). An advantage of using PCA as a pre-processing step is that components that do not describe large amount of variance are excluded early on (Thiebaut de Schotten et al., 2014), reducing the possible dimensionality of the data. Hierarchical clustering approaches (such as in Moreno-Dominguez et al., 2014) demonstrate results quite comparable to those shown here but at the cost of increasing complexity. Methods working in track space have been very efficient at clustering tractography streamlines themselves, and assigning anatomical labels to them with varying degrees of supervision, which then allows the labelling to be propagated to new subjects (O’Donnell and Westin, 2007, Tunç et al., 2014) but have not explicitly provided parcellations of grey matter. This method sits in the middle, a hybrid (O’Donnell et al., 2013), providing both a parcellation and an estimate of the anatomical course of a bundle.

In summary, we have used independent component analysis to reproducibly segregate whole brain grey matter according to diffusion tractography based white matter connectivity, providing anatomically meaningful networks of white matter connectivity. The approach distinguishes known, overlapping white matter pathways and shows good spatial correspondence to resting-state functional connectivity.

## Appendix A Supplementary material

.

Supplementary material **A note on format** Images in Supplementary Images 1–7 are cifti format files (allowing for mixed volume / surface data) in standard “greyordinate space” as in Glasser et al. (2013). They can be loaded in the connectome workbench image viewer: https://www.humanconnectome.org/software/connectome-workbench.html. Images in Supplementary Images 8 and 9 are nifti files in 2mm MNI space. Supplementary Images 1–5: Hard surface cortical parcellation of the Independent Component Analysis on the cortical surface for 5 different dimensionality of ICA: 50, 100, 150, 200 and 300. Supplementary Images 6 and 7: Raw and thresholded independent component analysis images in cifti format for the k=50 ICA. Supplementary Images 8 and 9: Raw and thresholded patterns of tractography derived from the independent component analysis in Supplementary Image 6. Images are ordered according to the ICA output in Supplementary Images 6 and 7.
