# Supplementary material for: Concurrent white matter bundles and grey matter networks using independent component analysis
Source: Neuroimage. 2018 Apr 15;170:296–306. doi: 10.1016/j.neuroimage.2017.05.012 (PMC6318261; doi:10.1016/j.neuroimage.2017.05.012)
Supplement: Supplementary file 1 — Supplementary material [file mmc1.docx]

**Appendix**

In the following, **C** is the **s** (seed) x **v** (voxel) tractography connectivity matrix. The matrix **C** is also standardised to 0 mean and unit standard deviation. This matlab pseudocode matches to Figure 2b and adapted directly from Smith et al (2014). The input matrix **C** can be from either a single subject or averaged across subjects.

dPCA=4000; %Dimensionality of the PCA

N=size(C,2); %Number of voxels total

order=randperm(N); %Randomise order for MIGP PCA

C_random=C(:,order);

n=10000; %Number of voxels to reduce at each iteration

W=[];

for i=1:n:N

data=C_random(:,i:min(i+n-1,N)); %Select data

W=[W; data];

[U,D]=eigs(W*W',min(dPCA,size(W,1)-1)); %Get the top eigenvectors of W

W=U'*W; %Multiply these into W to get weighted eigenvectors

end

data=W(1:dPCA,:); %Select the top eigenvectors

Next run the fastica algorithm (Hyvarinen, 1999) on the resulting PCA subspace. The algorithm below uses the implementation from <https://research.ics.aalto.fi/ica/fastica/> . data is the matrix **s** (seed) x **dPCA** from the MIGP

This matlab pseudocode matches to Figure 2c and 2d.

NComps = 50 %Dimensionality of the ICA and PCA fed into fastica

[icasig] = fastica (data, 'lastEig', NComps, 'numOfIC', NComps, 'approach', 'symm', 'g', 'pow3', 'finetune', 'pow3');

%Force the ica components to be positive on the long tail

for i=1:size(icasig,1)

    if ((prctile(icasig(i,:),1)+prctile(icasig(i,:),99.5))<0)

        icasig(i,:)=icasig(i,:)*-1;

    end

end

%Use linear regression of the spatial ICs (in seed space) to get their representation in tractography space

beta_tracks = pinv(icasig)***C**;
